# Knockdown resistance (*kdr*)-like mutations in the voltage-gated sodium channel of a malaria vector *Anopheles stephensi *and PCR assays for their detection

**DOI:** 10.1186/1475-2875-10-59

**Published:** 2011-03-14

**Authors:** Om P Singh, Cherry L Dykes, Manila Lather, Om P Agrawal, Tridibes Adak

**Affiliations:** 1 National Institute of Malaria Research, Sector 8, Dwarka, Delhi-110077, India; 2 School of Studies in Zoology, Jiwaji University, Gwalior-474011, India

## Abstract

**Background:**

Knockdown resistance (*kdr*) in insects, resulting from mutation(s) in the voltage-gated sodium channel (vgsc) gene is one of the mechanisms of resistance against DDT and pyrethroid-group of insecticides. The most common mutation(s) associated with knockdown resistance in insects, including anophelines, has been reported to be present at residue Leu1014 in the IIS6 transmembrane segment of the vgsc gene. This study reports the presence of two alternative *kdr*-like mutations, L1014S and L1014F, at this residue in a major malaria vector *Anopheles stephensi *and describes new PCR assays for their detection.

**Methods:**

Part of the vgsc (IIS4-S5 linker-to-IIS6 transmembrane segment) of *An. stephensi *collected from Alwar (Rajasthan, India) was PCR-amplified from genomic DNA, sequenced and analysed for the presence of deduced amino acid substitution(s).

**Results:**

Analysis of DNA sequences revealed the presence of two alternative non-synonymous point mutations at L1014 residue in the IIS6 transmembrane segment of vgsc, i.e., T>C mutation on the second position and A>T mutation on the third position of the codon, leading to Leu (TTA)-to-Ser (TCA) and -Phe (TTT) amino acid substitutions, respectively. Polymerase chain reaction (PCR) assays were developed for identification of each of these two point mutations. Genotyping of *An. stephensi *mosquitoes from Alwar by PCR assays revealed the presence of both mutations, with a high frequency of L1014S. The PCR assays developed for detection of the *kdr *mutations were specific as confirmed by DNA sequencing of PCR-genotyped samples.

**Conclusions:**

Two alternative *kdr-*like mutations, L1014S and L1014F, were detected in *An. stephensi *with a high allelic frequency of L1014S. The occurrence of L1014S is being reported for the first time in *An. stephensi*. Two specific PCR assays were developed for detection of two *kdr*-like mutations in *An. stephensi*.

## Background

*Anopheles stephensi *is one of the major vectors of malaria in India, Pakistan and Middle East and is regarded as an urban malaria vector [1]. This species is widely distributed in mainland India and its distribution extends beyond the west of India to Pakistan, Afghanistan, Iran, Iraq, Bahrain, Oman and Saudi Arabia, and in the east to Bangladesh, South China and Myanmar [2]. The control of this vector in India relies on indoor residual spraying (IRS) of insecticides in rural areas and anti-larval measures in urban areas. DDT is still the insecticide of choice for IRS in India and about 60% of the high-risk areas targeted under IRS are under the coverage of this insecticide [3]. Synthetic pyrethroids are alternative insecticides for use in IRS to control malaria vectors which are resistant to DDT and malathion [3]. These are the most effective insecticides for the control of malaria vectors and are regarded comparatively safer due to relatively low mammalian toxicity [4]. The use of pyrethroid-impregnated mosquito nets especially the long-lasting insecticidal mosquito nets (LLIN) is one of the cheapest and most effective interventions against malaria [5] and is being promoted in India with a target to cover 80% of population under the risk area having an API (annual parasite incidence, i.e., number of malaria cases per 1,000 persons per year) > 5 [3]. In urban areas where IRS is not feasible, such nets may provide effective protection against malaria. Another popular use of pyrethroids besides LLIN is in the form of commercial mosquito repellents such as mats, liquidators and coils.

Pyrethroids and DDT act on the insect's voltage-gated sodium channel (vgsc) proteins found in nerve cell membranes by altering the gating kinetics leading to paralysis and eventual death of the insect. Knockdown resistance is one of the mechanisms of resistance in insects against these insecticides, which is conferred by mutation(s) in the vgsc gene. Several such mutations reported in insects have been summarized by Davies & Williamson [6]. The most common mutation in insects, including anophelines, is leucine-to-phenylalanine change at residue L1014, commonly referred to as *kdr *(knockdown resistance) mutation. Variant mutations L1014S and L1014H have also been reported in insects; however, the latter has not been reported in anophelines. This paper reports the presence of two alternate mutations L1014S and L1014F in the Indian *An. stephensi *and describes development of PCR assays for genotyping both the *kdr*-like mutations.

## Methods

### Mosquito collection

*Anopheles stephensi *mosquitoes were collected from villages Akabarpur and Umran of Alwar district (Rajasthan, India), situated between 27°26' and 27°29'N latitude and between 76°31' and 76°35'E longitude. Two rounds of IRS of DDT is the current vector control measure being adopted in these villages. Adult females were collected from cattle-sheds using a mouth aspirator with the help of a flash torch. Some larvae were also collected from natural breeding habitats such as cement tanks and cisterns, and reared in insectarium till their emergence into adults. Larvae were reared in enamel trays measuring 20 × 30 cm containing water. Powdered fish-food and yeast powder were provided as food for larvae. Following pupation, pupae were picked up and placed in a plastic cup containing water which was kept inside a cloth cage measuring 30 × 30 × 30 cm for their emergence into adults. The temperature and relative humidity of insectary were maintained at 27°C and 70%, respectively. The adult mosquitoes were identified using keys by Christophers [7].

### DNA isolation

DNA was isolated from individual mosquitoes using the method by Livak [8]. Prior to DNA isolation, one-third of the lower-abdomen of female mosquitoes was removed in order to avoid DNA contamination originating from sperms of male sexual counterpart, stored in spermatheca.

### DNA sequencing of vgsc

**Figure 1:**
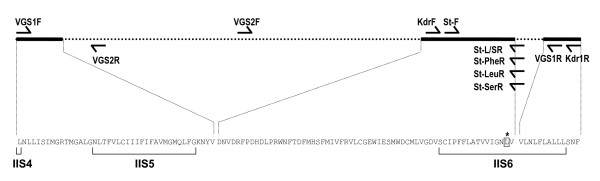
**Diagrammatic representation of a part of the voltage gated sodium channel sequenced and locations of primers used for sequencing and PCR assays**. Asterisk denotes amino acid position 1014 (the *kdr *locus), horizontal solid and dotted lines represent exons and introns, respectively, and harpoons represent primers.

Initially, a PCR fragment from IIS6 segment of the vgsc was amplified from two specimens of *An. stephensi *using primers KdrF (5'- GGA CCA YGA TTT GCC AAG AT -3') and Kdr1R (5'- TGG TGC AGA CAA GGA TGA AG -3') used for *Anopheles culicifacies *[9] in a reaction mixture (25 μl) that contained 1× Buffer, 1.5 mM of MgCl_2_, 200 μM of each dNTP, 0.5 μM of each primers and 0.625 unit of *Taq *DNA polymerase. The conditions of PCR were: an initial denaturation at 95°C for 5 min, followed by 35 cycles at 95°C for 30 S, 48°C for 30 S and 72°C for 45 S, and a final extension step at 72°C for 7 min. The PCR products were purified with QIAquick PCR purification kit (Qiagen) and were sequenced in both directions using BigDye V3.1 as per protocol provided by the vendor. Based on the sequences, a reverse primer VGS1R (5'- CGA AAT TGG ACA AAA GCA AGG -3') was designed for further amplifications. Subsequently, a 1.4 kb region encompassing IIS4-S5 linker-to-IIS6 transmembrane segment of vgsc was amplified using primers VGS1F (5'- CTG AAT TTA CTC ATT TCC ATC -3') and VGS1R. The reaction mixture (50 μl) contained 1× Buffer, 1.5 mM of MgCl_2_, 200 μM of each dNTP, 0.5 μM of each primers and 1.25 unit of *Taq *DNA polymerase. The cycling conditions were: an initial denaturation at 95°C for 3 minutes followed by 35 cycles, each consisting of denaturation at 95°C for 30 S, annealing at 55°C for 40 S and an extension at 72°C for 2 minutes, and a final extension at 72°C for 7 minutes. The PCR products were purified using Montage^® ^spin column (Millipore Corporation). Sequencing was performed by m/s Macrogen Inc, Korea using BigDye (Applied Biosystem) chemistry. Primer walking strategy was used for sequencing the product using primers VGS1F, VGS2R (5'- GAT ATG GTG CGA GCG AAT TT -3') and VGS2F (5'- ATC CGT TTG CCC AAA CTA CA -3'). The diagrammatic representation of location of primers used in this study is shown in Figure 1. All the sequences were assembled and the derived sequences of individual mosquitoes were submitted to GenBank (accession numbers: JF304952-to-JF304955).

For screening of *kdr *mutations in *An. stephensi *population, further sequencing was done using two separate PCRs, referred hereafter as PCR-1 and PCR-2, leaving most part of the intervening intron of 995 bp. For PCR-1 the primers used were VGS1F and VGS2R and for PCR-2, KdrF and VGS1R. The PCR conditions were same for both the PCRs. The reaction mixture (25 μl) contained 1× Buffer, 1.5 mM of MgCl_2_, 200 μM of each dNTP, 0.5 μM of each primers and 0.625 unit of *Taq *DNA polymerase (AmpliTaq Gold, Applied Biosystem). The conditions of PCR were: an initial denaturation at 95°C for 5 min, followed by 35 cycles at 95°C for 30 S, 50°C for 30 S and 72°C for 45 S, and a final extension step at 72°C for 7 min. The PCR products were purified with QIAquick PCR purification kit (Qiagen) and sequenced either at in-house sequencing facility using BigDye V3.1 or were done at Macrogen Inc, S Korea. Primers used for sequencing were VGS2R for PCR-1 and KdrF for PCR-2. A total of 22 samples were sequenced after amplification with PCR-1 and 39 samples following PCR-2.

### Statistical analysis

Hardy-Weinberg equilibrium (HWE) between different *kdr *genotypes was tested using software Arlequin ver 3.11 [10].

## Results

Analysis of DNA sequences revealed the presence of two alternative non-synonymous point mutations at L1014 residue in the IIS6 transmembrane segment of vgsc, i.e., T>C mutation on the second position and A>T mutation on the third position of the codon, leading to Leu (TTA)-to-Ser (TCA) and -Phe (TTT) amino acid substitutions, respectively. No other non-synonymous mutations were found in any other region of vgsc sequenced.

Visualization of PCR product amplified with PCR-2 revealed the presence of polymorphic amplicons of varying sizes. Analysis of DNA sequence chromatograms revealed the presence of a stretch of highly polymorphic microsatellite of CT-repeats downstream to *kdr *locus. The DNA sequencing chromatogram of microsatellite region was generally ambiguous due to polymorphism in size and pattern of sequence, and presence of stutter peaks.

### Development of PCR assays for *kdr *genotyping

**Figure 2:**
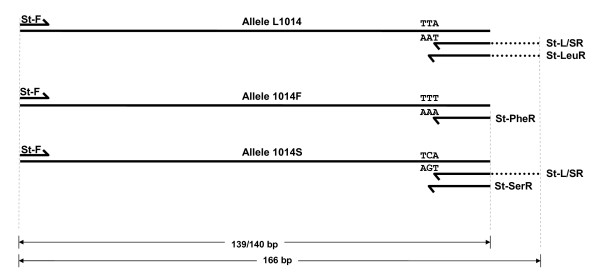
**Diagrammatic representation showing location of primers used in PCR developed for *kdr *genotyping, their annealing specificity with different *kdr *alleles and expected amplicon sizes**. Horizontal solid lines represent DNA templates with different alleles, harpoons represent primers and dotted lines represent primer-tail.

Designing of a classical tetra-primer Allele-Specific PCR (ASPCR) or an Amplification Refractory Mutation System (ARMS) for *kdr *genotyping in *An. stephensi *was not feasible due to the presence of highly polymorphic microsatellite located downstream to *kdr *locus that would lead to production of amplicons (containing microsatellite) of variable sizes making correct genotyping of *kdr *alleles difficult. Two PCR assays were designed for the genotyping of the *kdr *alleles wherein three primers were used in each PCR --a forward primer St-F (5'- GAT TGT GTT CCG TGT GCT GT -3') and two reverse allele-specific primers, all three located upstream to the microsatellite region. Since both the allele-specific primers designed for each PCR were from the same regions, a 26-bp tail [11] was added to the 5' end of one of the two allele-specific primers to differentiate the two alleles by the amplicons size. In the first PCR, hereafter called as PCR-F, the allele 1014F is discriminated from other alleles (wild and 1014S) and in the second PCR, hereafter called as PCR-L/S, 1014S and wild (L1014) alleles are discriminated. The allele-specific primers designed were: St-L/SR (5'- GCG GGC AGG GCG GCG GGG GCG GGG CCC GAT CGG AAA GTA AGT TAC TTA CG**t **CT -3') and St-PheR (5'- GAT CGG AAA GTA AGT TAC TTA CG**g **CA -3') for PCR-F, and St-LeuR (5'- GCG GGC AGG GCG GCG GGG GCG GGG CCC GAT CGG AAA GTA AGT TAC TTA CGA **g**TA -3') and St-SerR (5'- CGA TCG GAA AGT AAG TTA CTT ACG A**t**T G -3') for PCR-L/S. The primer St-L/SR was designed specific to both L1014 and 1014S alleles, St-PheR to 1014F, St-LeuR to L1014 and St-SerR to 1014S allele. The diagrammatic representation of annealing specificity of each allele-specific primer to specific template DNA is shown in Figure 2. A tail of 26 bp was incorporated in primer St-L/SR and St-LeuR (shown underlined in corresponding primer sequences) following Saavedra-Rodriguez *et al *[11]. To prevent non-specific annealing, an additional mismatch was incorporated on the 3^rd ^base from the 3' end in each of the allele-specific primers, which are shown in lower case in corresponding primer sequences. The expected amplicon sizes formed by the allele-specific primers St-L/SR and St-PheR (with primer St-F) in PCR-F are 166 and 139 bp receptively. The expected size of amplicons in PCR-L/S with allele-specific primers St-LeuR and St-SerR are 166 and 140 bp, respectively.

The optimized PCR conditions for PCR-F were as follows. The PCR was carried out in 15 μl reaction volume containing 0.50 μM of St-PheR, 0.25 μM of St-L/SR and 0.25 μM of St-F, 1× buffer, 1.5 mM of MgCl_2_, 200 μM of each dNTP and 0.375 units of *Taq *DNA polymerase (AmpliTaq Gold, Applied Biosystem). The PCR thermal cycling conditions were: one cycle at 95°C for 5 min; followed by 35 cycles each at 95°C for 30 S, 55°C for 30 S and 72°C for 45 S, and a final extension step at 72°C for 7 min. The amplified products were eletrophoresed on 3.0% agarose gel containing ethidium bromide and visualized under UV illumination in gel documentation system. The presence of 139 bp product was scored as 1014F allele and 166 bp that of the other alternative alleles (L1014/1014S). Presence of 139 bp and absence of 166 bp was scored as homozygous 1014F.

The PCR conditions for PCR-L/S were similar to PCR-F except for primers concentration which is 0.50 μM for all the primers (St-F, St-L/SR and St-SerR). The presence of 166 bp PCR band was scored as wild allele (L1014) and 140 bp as 1014S allele. No band was expected for homozygous 1014F in this PCR; it is, therefore, not necessary to run PCR-L/S for samples scored as homozygous 1014F in first PCR, i.e., PCR-F.

**Figure 3:**
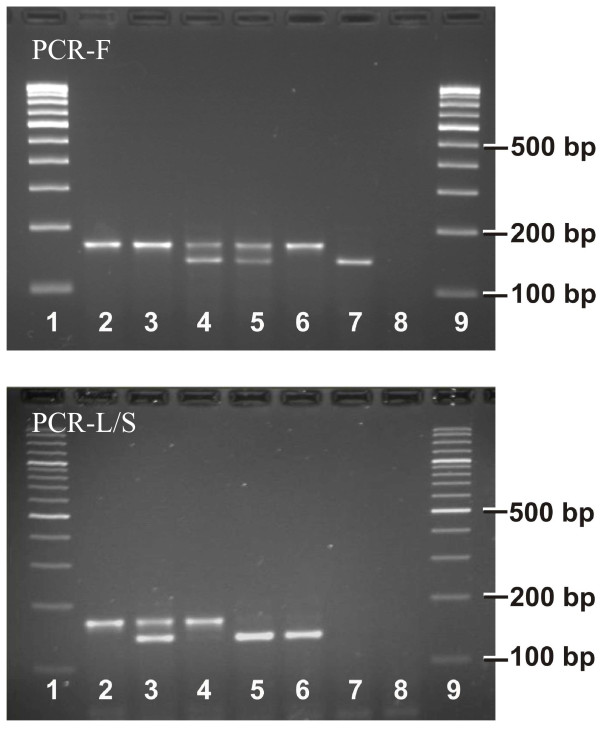
**Gel photographs showing result of PCR-F and PCR-L/S**. Lanes 1 and 9: 100 bp DNA ladder; Lane 2: L/L; Lane 3: L/S; Lane 4: L/F; Lane 5: F/S; Lane 6: S/S; Lane 7: F/F (sample collected from Delhi, India); Lane 8: negative control. The letters L, S and F stands for leucine, serine and phenylalanine, respectively.

**Figure 4:**
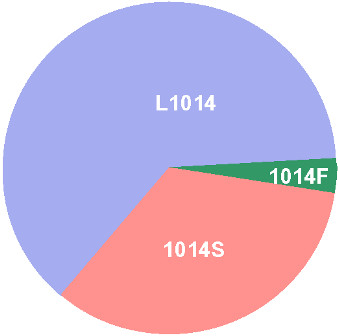
**Pie diagram showing allelic frequencies of L1014, 1014F and 1014S alleles in Alwar**.

The two PCR assays developed for identification of the three alleles at position 1014 of vgsc provided discrete bands as expected (Figure 3). A total of 121 mosquito individuals were genotyped using PCR-L/S and PCR-F. The numbers of samples with different genotypes were as follows: L/L = 45 (37.2%), L/S = 55 (45.5%), L/F = 6 (5%), S/S = 12 (9.9%), FF = 0 and S/F = 3 (2.5%). The allelic frequency of the 1014S and 1014F were 0.339 and 0.037, respectively (Figure 4). The different genotypes were in HWE (*p *= 0.8039)

The result of PCR-genotyping of samples, which were sequenced for IIS6 transmembrane segment (following PCR-2, n = 39) were in agreement with sequencing results.

## Discussion

Knockdown resistance in insects against insecticides DDT and pyrethroids is conferred by one or more mutations present in the target site, the vgsc. Of the several *kdr*-like mutations reported in insects, mutations at residue L1014 leading to Leu-to-Phe substitution is most common, known in at least two dozen of insect species [6]. Variant mutations at this residue, i.e., Leu-to-Ser/His [6] have also been reported. In anophelines, the most common mutation conferring knockdown resistance is L1014F, first reported in *Anopheles gambiae *[12] and subsequently in *Anopheles sacharovi *[13], *An. stephensi *[14], *Anopheles arabiensis *[15], *An. culicifacies *[9] and several other anophelines [16]. Another alternative mutation, L1014S, which was reported in *An. gambiae *[17], *An. Arabiensis *[18] and *An. sacharovi *[13] only prior to the year 2010, has now been reported in six more anopheline species, viz. *An. culicifacies *[19], *Anopheles vagus, Anopheles sinensis, Anopheles paraliae, Anopheles peditaeniatus *[16] and now in *An. stephensi *(this study). Both these *kdr *mutations have been reported to confer pyrethroid resistance at least in *An. gambiae *[20-23]. The conserved nature of L1014F and L1014S mutations in several insects indicate their possible role in phenotypic knockdown resistance in other insects too.

Sequencing of part of vgsc (spanning IIS4-S5 linker-to-IIS6 transmembrane) of wild *An. stephensi *revealed the presence of two alternative mutations--L1014F and L1014S in the IIS6 transmembrane segment. No other mutation was recorded in the region sequenced. Earlier, presence of only one mutation L1014F was reported in *An*. *stephensi *[14,24] while the presence of L1014S in *An. stephensi *is being reported for the first time. Limited data generated from one locality in this study reveals predominance of L1014S mutation; however, a thorough survey is required to map the distribution of the two mutations in different eco-epidemiological paradigm with different insecticide selection pressure.

Monitoring of insecticide resistance in vector population is an essential component of any malaria control programme for eliciting effective decision making process in the selection of vector control options. For the identification of *kdr *mutations, PCR-based methods have been widely used which are simple and cost-effective. Allele-specific PCR is the most common method employed but may not prevent mispriming because allele-specific primers are based on single base primer-template mismatch [25]. Alternative PCR-based methods such as ARMS, a variant of ASPCR where an additional mismatch is incorporated near the 3' end of allele-specific primers to increase their specificity [26], and PIRA-PCR (primer-introduced restriction analysis-PCR) are highly specific but have been rarely used for *kdr *analysis [9,19,27]. Earlier, an ASPCR strategy was developed by Enayati *et al *[14] for genotyping L1014F mutation in *An. stephensi *and was successfully used for genotyping of Iranian *An. stephensi*. However, the same PCR will have limitation with Indian *An. stephensi *population due to the presence of highly polymorphic (in length) microsatellite in the amplicons resulting in variable sizes of PCR products. Furthermore, the presence of two bands of variable sizes for a specific allele in case the sample is heterozygous for microsatellite repeats of different lengths, renders tetra-primer strategies (ASPCR or ARMS) unsuitable for Indian population. Therefore, alternative strategies for genotyping of 1014F and 1014S were designed wherein all the primers used in the PCR strategies are from the region upstream to microsatellite locus. To prevent non-specific annealing, an additional mismatch has been incorporated in all the three allele-specific primers on the 3^rd ^base from the 3' terminus, based on the principle of ARMS.

Unlike a conventional tetra-primer ASPCR or ARMS, where a common PCR band is formed by a pair of external primers flanking mutation site and which serves as an internal control, there is no such control band in PCRs developed in this study. Absence of such a band in ASPCR or ARMS indicates PCR failure or presence of null allele. The PCR strategies developed in this study are so designed that PCR-failure or presence of null alleles can be determined without the control band. Since at least a single band is expected to be formed during PCR-F in all samples with every possible genotypic combination, the absence of both the bands in PCR-F may be considered as PCR failure or presence of null allele. Likewise in PCR-L/S, at least one band will be seen in all genotypes except in the case of homozygous 1014F (which is not required to be analysed with PCR-L/S). Therefore, absence of band in PCR-L/S (except for homozygous 1014F samples) may be considered as a case of PCR-failure or presence of null allele. In such cases it is recommended that PCR should be repeated and the quality of PCR be checked. However, in the present study no null allele was noticed.

There are several techniques available in literature for *kdr *genotyping which have been discussed by Bass *et al *[28], Janeira *et al *[27] and Sarkar *et al *[29]. Real-Time fluorescent-based methods like Fluorescence Resonance Energy Transfer/Melt Curve analysis (FRET/MCA) [18], single-labelled hybridization probe/melting curve analysis [29] and TaqMan^® ^[28], which are claimed to be highly sensitive, requires expensive and sophisticated real-time PCR machine and labelled probes/primers. The commercial cost of such labelled probes/primers is high due to modifications and post-synthesis HPLC or gel purification involved. A cost effective Real-Time technique used for *kdr *genotyping is High Resolution Melt (HRM) analysis [28], which is based on the differences in melting profile of PCR amplicons with different *kdr *genotypes. This technique is not very promising for *An. stephensi *due to additional (synonymous) SNP(s) present in the flanking regions of *kdr *locus which may alter the melting profile of an amplicon with a particular *kdr *genotype. Simple PCR assays, on the other hand, are cheap alternatives which require less sophisticated and easily accessible low-cost equipments--a thermal cycler, a gel electrophoresis system and an UV-transilluminator. In the PCR assays developed in this study, a mismatch was introduced in allele-specific primers near the 3' end as this tremendously increases the specificity of primers [25]. These PCR assays were specific and can be performed in most of the laboratories equipped with basic PCR facilities. Several other techniques for *kdr *genotyping such as Heated Oligonucleotide Ligation Assay (HOLA) [30], Sequence Specific Oligonucleotide Probe - Enzyme-Linked ImmunoSorbent Assay (SSOP-ELISA) [31] and PCR-Dot Blot, where initially PCR product is amplified using primers flanking *kdr *locus and subsequently subjecting it to special treatments for *kdr *genotyping, are relatively time consuming due to lengthy procedures involved [29].

## Conclusions

The present study reports the presence of two alternative *kdr-*like mutations in IIS6 transmembrane segment of vgsc, i.e., L1014S and L1014F, in *An. stephensi *collected from Alwar district of Rajasthan, India. The L1014S mutation was predominant with an allelic frequency of 0.339 and its presence is being reported for the first time in *An. stephensi*. The allelic frequency of other mutation, L1014F, was 0.037. Specific PCR assays were developed for detection of both the *kdr*-like mutations in *An. stephensi*.

## Competing interests

The authors declare that they have no competing interests.

## Authors' contributions

OPS designed the study, analysed sequences, designed PCR strategies, and wrote the manuscript; CLD and ML did optimization of PCR and performed genotyping, OPA and TA contributed to the manuscript. All authors provided critical reviews of the manuscript and approved the final version.
